# The impact of COVID-19 pandemic on the healthcare workers mental health

**DOI:** 10.1192/j.eurpsy.2023.891

**Published:** 2023-07-19

**Authors:** M. Ferrandino, V. Sollo, M. Di Vincenzo, N. Marafioti, B. Della Rocca, C. Brandi, V. Giallonardo, M. Luciano, G. Sampogna, A. Fiorillo

**Affiliations:** 1 Psychiatry, University of Campania “L. Vanvitelli”, Naples, Italy

## Abstract

**Introduction:**

The COVID-19 pandemic represents an unprecedented in health events that has had a negative impact on the mental health of the population in general as well as on specific categories, including patients with mental and physical disorders, and healthcare professionals. In particular, COVID-19 pandemic has produced extraordinary stress in healthcare workers, especially frontline physicians, nurses and healthcare professionals.

**Objectives:**

In the present study we aimed to evaluate levels of burnout, a clinical condition characterized by emotional, psychological and physical exhaustion, in a sample of health workers from the Campania region, Italy, during the first phase of the COVID-19 pandemic. Secondary objectives of the study include the assessment, in the same group, of levels of anxiety-depressive symptoms, insomnia, suicidal ideation and symptoms on the post-traumatic spectrum.

**Methods:**

An online survey was released through the official website of the University of Campania “L. Vanvitelli” and social media. The Maslach Burnout Inventory was used to assess burnout in the healthcare professionals; Depression Anxiety Stress Scale-21 Short Version to measure levels of anxiety, depression and stress; the Insomnia Severity Index was used to identify insomnia-related symptoms; the Suicidal Ideation Attributes Scale was adopted to select individuals based on the presence of suicidal thoughts while the Impact of Event Scale-Revised was administered to evaluate trauma-related dimensions.

**Results:**

A total of 389 health workers was recruited. They were predominantly female, with an average age of 39.06 (± 11.85) years, working mainly in the second line hospitals during the COVID-19 emergency. During the pandemic, first- or second-line health workers reporting significant levels of emotional exhaustion are on average 23,89 (±4.22), those reporting feelings of depersonalization are on average 7.58 (±2.73), while those who report a good level of professional efficiency are on average 21.12 (±3.48).

Predictors of increased levels of depersonalization are being a first-line worker and the presence of traumatic event avoidance symptoms. Furthermore, levels of professional fulfillment are negatively affected by age, the presence of intrusive symptoms, the presence of sleep disorders, and being a frontline worker.

**Conclusions:**

The impact of the COVID-19 pandemic on the mental health of healthcare professionals involved in the first and/or second line COVID hospitals is indisputable. Although burnout syndrome is not a new clinical condition, the COVID-19 pandemic may further worsen the magnitude of the problem. However, our results could be a starting point to promote a change in the way we perceive the mental health of healthcare professionals.

**Disclosure of Interest:**

None Declared

